# Supplementary material for: Insecticide resistance in Aedes aegypti from Tapachula, Mexico: Spatial variation and response to historical insecticide use
Source: PLoS Negl Trop Dis. 2021 Sep 27;15(9):e0009746. doi: 10.1371/journal.pntd.0009746 (PMC8475978; doi:10.1371/journal.pntd.0009746)
Supplement: Attachment — Submitted filename: Responses to review comments_1.docx [file pntd.0009746.s003.docx]

**Responses to review comments (PNTD-D-20-02240)**

**Methods**

**Study Design**

**Critiques:**

Reviewer 1:

- Line 153: “Females were bloodfed to obtain the F1 generation.” Please specify the animal species on which the females were bloodfed.

*That phrase was changed to: “Females were bloodfed from rabbit to obtain the F_1_ generation.”*

- Lines 198-199: The authors describe the use of city quadrants as the basis for some of their spatial variation analysis, however this seems like a somewhat arbitrary geographical division on which to perform spatial analysis. For instance, two collection sites may be very close in linear distance, but are located in different quadrants, leading to underestimation of spatial effects between quadrants. While it is okay to keep the quadrant spatial analysis in the paper, I recommend they discuss the limitations of this approach in more detail in the Discussion section.

*We agree, we have described the experimental design in the methodology (line 133) adding: “We tested 26 collection sites located in the city of Tapachula. Each collection site consisted in nine blocks and these were selected based on vehicle access for outdoor spraying. To minimize the effects of mosquito migration by flight range (50 - 150 m), sites were located at least 250 m apart. The spatial correlation between resistance and geographical distance was calculated for the 26 collection sites. In addition, since Tapachula’s vector control program uses a quadrant subdivision for spraying activities, we included a second analysis to test this source of variation by assigning sites to one of the four cardinal geographical quadrants (NE, NW, SE, and SW).”*

- Lines 200-207: The authors describe a correlation analysis (using Moran’s correlograms) testing the hypothesis that mosquitos from neighboring collection sites will have more similar LC50s than mosquitos from distant sites, however I do not see the results of this analysis in the Results or Discussion section. Please include this data in the revised manuscript (even if the results are negative or placed in Supplemental Data), as they provide more robust and meaningful spatial information than the quadrant-based approach.

*We agree, we have included in the methodology (line 199) changing the whole paragraph to: “To test the hypothesis of resistance correlation with space, we created Moran’s I correlograms as implemented in PASSAGE 2.0 [20]. Mosquitoes from different collection sites were considered neighbors if the sites were within 250 meters of each other. We expected that the LC_50_s or kdr frequencies would be associated with geographical distance (i.e., that closer neighbor sites would show similar resistance levels, compared to those farther away). A second analysis to test the variation of the LC_50_s and kdr frequencies between and within quadrants using a linear regression model and ANOVA in R (car package) was conducted. Since the city is uniformly sprayed during a cycle, we did not expect variation between or within quadrants. Correlation between kdr frequencies and LC_50_’s for permethrin and deltamethrin was tested using a Spearman test.*

*In the results section (line 277) we will include the correlogram figure and the following statement: “To assess the correlation of LC_50_’s with space, we generated Moran’s I correlograms for each of the five insecticides (Fig 4). The analysis included all 26 collection sites. We did not detect a discernable pattern in any of the tested insecticides. We expected a positive correlation (Moran’s I statistically > 0) between nearby sites, then as the distance increased (between the samples) the correlation would decrease, and later would turn negative (Moran’s I statistically < 0). However, this was not observed. Although, few of the distance classes where statistically different from zero (eg. bendiocarb at 3250 m, 3750 m, 4750 m, and 6250 m; malathion at 1500 m; and deltamethrin at 3750 m, and 4250 m). A caveat in our analysis is the possibility that there is autocorrelation at smaller distances than the ones we selected (x < 250 m). Our experimental design was not geared towards the detection of spatial correlation at smaller distance; there is very small number of samples below 250 meters.”*

Reviewer #3

I had three specific critiques of the methods:

-Line 140: What was the material and color of the ovitraps used? Where they a specific brand or homemade?

*We have added the next description within line 140: “Ovitraps were made by hand with transparent, inert, non-toxic polypropylene (PP) cups of a 1 L capacity, and painted black on the outside following the guidelines for Entomological Surveillance with Ovitraps of the Mexican Ministry of Health (15).”*

-Line 146: At what approximate concentration (grams of diet per pan, etc.) and at what frequency were the larvae fed?

*The phrase was corrected at the end of the paragraph: “On the third and sixth day, the hatched larvae were fed a diet of Harlan® 5001 proteins, with 0.4 gr or 0.8 gr / 1.2 L for 1^st^-2^nd^ stadium and 3^rd^-4^th^ stadium at the 3^rd^ and 6^th^ day, respectively.”*

-Lines 165-175: What was the control treatment used for the LC50 testing? Was acetone alone used? Were the control mosquitoes untreated? Both? Please clarify.

*We added at the end of the paragraph (line 175): “As control, a bottle impregnated only with acetone was used each time an insecticide test with field or susceptible mosquitoes was run.”*

**Results**

Reviewer #1

-The analysis presented does not match the stated analysis plan, as I do not see any results for correlating LC50 profile similarity with distance to other collection sites (Lines 200 to 207), as discussed above.

*We agree, we have included the Moran’s I correlogram Figure (Fig 4) and results description (line 277) as follow:*

*“To assess the correlation of LC_50_’s with space, we generated Moran’s I correlograms for each of the five insecticides (Fig 4). The analysis included all 26 collection sites. We did not detect a discernable pattern in any of the tested insecticides. We expected a positive correlation (Moran’s I statistically > 0) between nearby sites, then as the distance increased (between the samples) the correlation would decrease, and later would turn negative (Moran’s I statistically < 0). However, this was not observed. Although, few of the distance classes where statistically different from zero (eg. bendiocarb at 3250 m, 3750 m, 4750 m, and 6250 m; malathion at 1500 m; and deltamethrin at 3750 m, and 4250 m). A caveat in our analysis is the possibility that there is autocorrelation at smaller distances than the ones we selected (x < 250 m). Our experimental design was not geared towards the detection of spatial correlation at smaller distance; there is very small number of samples below 250 meters.*

***Fig 4. Moran’s I correlograms as implemented in PASSaGE 2.0 assessing the correlation of LC****_50_****’s with space for permethrin (pyrethroid), deltamethrin (pyrethroid), chlorpyrifos (organophosphate), malathion (organophosphate), and bendiocarb (carbamate)****. The analysis included 26 collection sites in Tapachula, Chiapas, Mexico. Aedes aegypti mosquitoes from different collection sites were considered neighbors if the sites were within 250 meters of each other.”*

Reviewer #2

-Line 260. Table 3. It would be useful to describe the mutation status per individual mosquito (raw data as supplement) and a summary in the results for the two mutation sites. It is not likely that the mutations are evolving or acting independently.

*A S2 Table was add as supplemental data with results of individual mosquitoes, and a summary of the total genotype counts and allele frequencies in the completely collected area from Tapachula, as well as per each quadrant were added in the table 3*. *About the unlikely of mutations being evolving or acting independently is explained on the discussion section.*

-Line 269 - What is known about these particular mutations and insensitivity towards Type I vs Type II pyrethroids?

W*e added the phrase in line 272 “Although it is known that C1534 shows protection only against deltamethrin (12*)”

-Line 275 - The significance is said to have disappeared, but the sentence is ambiguous and the reader cannot tell whether this is just for the deltamethrin analysis or the permethrin analysis as well. This is clarified in the Discussion, but it would be better to clarify at this first instance where it is mentioned.

*We added in that paragraph (line275): “for both insecticides”*

-Line 337 - Target-site mutations (acetylcholinesterase) are known to be unlikely in *Aedes aegypti*, so this is not an unexpected result. A reference about this should be included here.

*We added at the end of the paragraph: “that in fact no mutations have been found in ace-1* *gene in* Aedes aegypti *[35].”*

-Line 352 - No geographical correlation with resistance patterns were found. Is anything known about the genetic population structure or history of colonisation by *Ae. aegypti* in Mexico? Resistance alleles can be brought in with mosquitoes invading from other places. Is this a likely scenario in Tapachula? In some cases it might explain the presence of resistance where there is no local history of use of a particular insecticide in the local area.

*We agree, we have added in the discussion “A previous study used mitochondrial ND4 haplotypes to determine gene flow patterns among 38 Ae. aegypti coastal collections in Mexico (37). Three genetic clusters were identified, the Northeast, Pacific, and Yucatan peninsula. For all sites, genetic distances remained small below geographic distances of 90 km and became large at distances >150 km. The Pacific cluster had the highest gene flow and diversity. A second study in the Yucatan Peninsula showed high gene flow occurring across 27 Ae. aegypti collection sites located up to 150 km of distance. Single nucleotide polymorphism (SNPs) at eleven loci did not vary across sites, suggesting high levels of gene flow. In contrast, insecticide resistance loci, including kdr alleles (I1016 and C1534) were highly variable across sites, indicating that insecticide resistance offsets the homogenizing effects of gene flow (38). In this study, we assume complete gene flow among collection sites because: 1) Tapachula belongs to the Pacific cluster, 2) Ae. aegypti is well established throughout the year and, 3) collection sites are within 10 km of distance. However, this remains to be tested.”*

Reviewer #3

I have a specific critique of Figure 1:

-Please clarify the location of ALL the sites by placing a dot indicating each site. Some sites (e.g. Raymundo Enriquez, Paraiso, etc.) have the resistance ratios placed adjacent to the site, but the site location is not clear.

*Corrected*

**Conclusions**

Reviewer #1

-Please address the limitations of the quadrant-based spatial analysis, as detailed above.

*We agree, we have added (line 365) “Two different analysis were conducted 1) the spatial analysis included all 26 sites and, 2) the quadrant analysis to identify operational sources of heterogeneity. The quadrant analysis doesn’t include a geographical component and has limitations.”*

Minor points to clarify:

- Discuss the possible contribution of regional/spatial heterogeneity in the intensity/frequency or type of insecticide used in Tapachula that can explain the spatial variation in resistance profiles. It sounds like insecticides were used uniformly throughout the city, adhering to national guidelines, but it would be helpful for the authors to include these potential contributing factors in the Discussion.

*We agree, we have added (line 346) “During vector control programs, the city of Tapachula is uniformly sprayed, using the same insecticide, frequency and intensity. More yet, we selected sites based in their accessibility to spraying-vehicles. Assuming that no spatial heterogeneity in frequency and intensity of spraying, we did not expect the high levels of variation in resistance profiles across de city. For example, significant heterogeneity in the frequency of kdr haplotypes was detected in Ae. aegytpi collected between city blocks in a town of Yucatan, suggesting that selection for these haplotypes occurs at a fine spatial scale (35). However, in contrast to our study, insecticide application was highly variable in space and time, creating a mosaic of selection pressures. In our study, some sources of heterogeneity could occur from mosquito migration from untreated sites due to vehicle inaccessibility, including parks, cemeteries, steep and unpaved streets. A second source of insecticide pressure is by use of household aerosol insecticides. For example, in a previous study from Merida, Mexico approximately 87% of households used commercially available pyrethroid products to control mosquitoes in their homes. Future studies should include an assessment of this source of selection pressure in Tapachula.”*

- Discuss how much population mixing you would expect between *Aedes* in neighboring collection sites. How frequently and how far does *Aedes* tend to migrate between different areas in urban habitats? How does that inform how you interpret the spatial variation you observe in your phenotypic and genotypic data?

*We agree, see answer to reviewer 2 above (line 352): “A previous study used mitochondrial ND4 haplotypes to determine gene flow patterns among 38 Ae. aegypti coastal collections in Mexico (37). Three genetic clusters were identified, the Northeast, Pacific, and Yucatan peninsula. For all sites, genetic distances remained small below geographic distances of 90 km and became large at distances >150 km. The Pacific cluster had the highest gene flow and diversity. A second study in the Yucatan Peninsula showed high gene flow occurring across 27 Ae. aegypti collection sites located up to 150 km of distance. Single nucleotide polymorphism (SNPs) at eleven loci did not vary across sites, suggesting high levels of gene flow. In contrast, insecticide resistance loci, including kdr alleles (I1016 and C1534) were highly variable across sites, indicating that insecticide resistance offsets the homogenizing effects of gene flow (38) In this study, we assume complete gene flow among collection sites because: 1) Tapachula belongs to the Pacific cluster, 2) Ae. aegypti is well established throughout the year and, 3) collection sites are within 10 km of distance. However, this remains to be tested.”*

Reviewer #2

-It would be useful to discuss the VGSC results in light of the hypotheses of co-evolution of Ile1016 and Cys1534 in Mexico put forward by Vera-Maloof et al. 2015 (PlosNTD).

*We agree, we added the next paragraph in the discussion (line 321): Historical data of kdr mutations indicated that C1534 confers low level of resistant on its own, and that resistance increased dramatically when I1016 evolved from the V1,016/C1,534 haplotype in field mosquito collected in different places from Mexico (33). Those results demonstrated that I1016 was unlikely to have evolved independently, and that both mutations need coexist in the same mosquito in order to confer higher levels of resistance.*

Editorial and Data Presentation Modifications?

Reviewer #2

-Zika should be written with a capital Z as it is named after Zika forest.

*We do not agree, since the capital letter is used only when the disease or virus refers that it is from the Zika place (proper name). Example: Zika virus, Zika disease, but not when is used informally as simply “zika” as it is written all three times in our manuscript.*

-Page 7 - Line 128 - remove apostrophe from OP's. It is better written as OPs (no possessive required).

*Corrected*

Reviewer #3

My editorial suggestions:

Minor edits in the Introduction section:

-Lines 110-111: Knockdown resistance is defined, however, I think it would be useful to also indicate that knockdown is a characteristic effect of the pyrethroid insecticide class.

*There, it already says "insects are not knocked down immediately after exposure to pyrethroids"*

-Line 128: Remov*e* the apostrophe for OPs (should be OPs, not OP's)

*Corrected*

Minor edits in the Methods section:

-Line140: What was the material and color of the ovitraps used? Where they a specific brand or homemade?

W*e have added the next description within line 140: “Ovitraps were made by hand with transparent, inert, non-toxic polypropylene (PP) cups of a 1 L capacity, and painted black on the outside following the guidelines for Entomological Surveillance with Ovitraps of the Mexican Ministry of Health (15).”*

-Line 146: At what approximate concentration (grams of diet per pan, etc.) and at what frequency were the larvae fed?

*The phrase was corrected at the end of the paragraph: “On the third and sixth day, the hatched larvae were fed a diet of Harlan® 5001 proteins, with 0.4 gr or 0.8 gr / 1.2 L for 1^st^-2^nd^ stadium and 3^rd^-4^th^ stadium at the 3^rd^ and 6^th^ day, respectively.”*

-Lines 165-175: What was the control treatment used for the LC50 testing? Was acetone alone used? Were the control mosquitoes untreated? Both? Please clarify.

*We added at the end of the paragraph (line 175): “As control, a bottle impregnated only with acetone was used each time an insecticide test with field or susceptible mosquitoes was run.”*

-Line 184: The second H null here is written as 'Ho', please correct to H null similar to elsewhere in the paper.

We agree, we changed the phrase line 183-184 to “to accept the Ho”

Minor edit to the Results section:

-Line 234: Please correct 'RR ... is shown' to 'RRs ... are shown'.

*Corrected*

Minor edit to the Discussion section:

-Line 282: Please correct '[2014]' to '[21]'.

*Corrected*

Minor edit to Figure 1:

-Please clarify the location of ALL the sites by placing a dot indicating each site. Some sites (e.g. Raymundo Enriquez, Paraiso, etc.) have the resistance ratios placed adjacent to the site, but the site location is not clear.

*Corrected*

**Minor**

Summary and General Comments

Reviewer #1

-The main weakness in the study is the absence of determining the relationship between distance of neighboring collection sites and insecticide resistance profile similarity, i.e. if neighboring mosquitos have more similar resistance phenotypes/genotypes than spatially distant non-neighbors. Related to this criticism is the use of a quadrant-based system to determine spatial effects, which has methodological limitations.

*We disagree, the spatial analysis did not include the quadrant subdivision, we used all collection sites. However, we were not clear in explaining the different analysis. Now we include a statement showing our reasons for the quadrant analysis and the spatial analysis using all collection sites.*

Reviewer #2

-The paper is clearly written and the experimental approach seems rigorous. A little more attention to the published literature at the points mentioned above would improve the manuscript.

*Comments from four references were added in the discussion and therefore into the references seccions.*

**Changes that we did to improve the manuscript:**

Commas were changed by semicolons in the Keywords.

Since most of the time was used pyrethroid, organophosphate and carbamate in the manuscript, PYR, OP, and CARB were deleted or changed to pyrethorid, organophosphate and carbamate.

Line 89, “(OPs)” was deleted.

Line 90, “(PYRs)” was deleted.

Line 96, “OPs” was changed to “organophosphates”

Line 97, “(CARBs)” was deleted.

Line 100, “PYR” was changed to “them”

Line 127, “PYR” was changed to “pyrethroids”

Line 128, “OPs” was changed to “organophosphates” and “CARB” was changed to “carbamate”.

Line 179, “PYRs = pyrethroids, OPs = organophosphates, CARBs = carbamates.” was added to the end of the table.

Line 234 “OPs” was changed to “organophosphates” and “CARBs” was changed to “carbamates”.

Line 246 and 247, “OP” was changed to “organophosphate” and “CARB” was changed to “carbamate”.

Line 282, “PYRs” was changed to “pyrethroids”

Line 293, “OPs” was changed to “organophosphates”.

Lines 311, 312 and 368 “PYRs” and “PYR” were changed to “pyrethroids”.

Line 367, “OPs” was changed to “organophosphates” and “CARB” was changed to “carbamate”.

Line 146, “Harlan®” was changed to “Harlan”.

Line 159, “Sigma®” was changed to “Sigma”.

Line 257 was changed from: “Except for NE-2 and SW-4, the genotype frequencies at the V1016I loci were in HW equilibrium at 24 of the 26 sites.” to “Except for NE-2 and SW-4, the genotype frequencies at the V1016I loci were in HW equilibrium.”

Line 267, we added “While NE-7 was calculated with an intermediate value of 0.61.”

Table 3 was corrected, since in addition to the subtotals and the total that were added within the same table, six errors were also found in the numbers, so they were changed. The asterisks indicating that the data at the F1534C are not in Hardy Weinberg equilibrium were also corrected. Therefore we made the next changes:

Line 258, the phrase “at 24 of the 26 sites” was removed since we detected a pleonasm.

Line 261, the phrase “from *Aedes aegypti* collected” was added to complete the description in this table.

Within the table “F1534 genotypes” changed to “F1534C genotypes”.

Line 267, the phrase “While NE-7 was calculated with an intermediate value of 0.61” was added.

Line 297, “A previous survey performed in Chiapas in 2016–2017 showed permethrin RRs > 50-fold and deltamethrin RRs >100-fold at 13 of 16 sites (unpublished data). Thus, possibly, the mosquito populations in Tapachula are undergoing a process of declining PYR resistance that will take several years.”

was changed to:

“Currently, we are conducting a study to determine the degree of loss of resistance to pyrethroids from 2016 to 2020 in this study area, which will demonstrate whether mosquito populations in Tapachula are undergoing a process of decreasing resistance that will take several years.”

Line 372, we added the acknowledgments as follow: “Acknowledgments The authors would like to thank Biol. Elsa Contreras, Geovanni Vázquez and Francisco Pozos from the Programme of Vector Control in Tapachula for contributions in the field work.”

References 11, 15, and 17 were corrected.

Each reference was checked and actualized with a link where the paper is available.

Line 527, Table S1, the word “Mexico” was added to the end of the main title.

We added the next paragraph at the end of the manuscript:

**“Table S2. Genotype for two *kdr*-associated substitutions (V1016I and F1534C) per individual *Aedes aegypti* mosquito (n = 47 to 50) at 26 sites in Tapachula, Chiapas, Mexico.** For V1016I: AA = homozygote for Ile_1016_, resistant; GG = homozygote for Val_1016_, susceptible; AG = heterozygote Ile_1016_/Val_1016_. For F1534C: TT homozygote for Phe_1534_, susceptible; GG = homozygote for Cys_1534_, resistant; TG = heterozygote Phe_1534_/Cys_1534._”
